# Supplementary material for: Recurrent SARS-CoV-2 infections and their potential risk to public health – a systematic review
Source: PLoS One. 2021 Dec 9;16(12):e0261221. doi: 10.1371/journal.pone.0261221 (PMC8659325; doi:10.1371/journal.pone.0261221)
Supplement: S1 Table — (DOCX) [file pone.0261221.s002.docx]

**Table S 1. Search Strategy**

| Pubmed | ((("Severe Acute Respiratory Syndrome Coronavirus 2" [Supplementary Concept] OR "COVID-19" [Supplementary Concept] OR "covid 19 diagnostic testing" [Supplementary Concept] OR "covid 19 drug treatment" [Supplementary Concept] OR "covid 19 serotherapy"[Supplementary Concept] OR "covid 19 vaccine" [Supplementary Concept] OR "Severe Acute Respiratory Syndrome Coronavirus 2"[tiab] OR ncov*[tiab] OR covid*[tiab] OR sars-cov-2[tiab] OR "sars cov 2"[tiab] OR "SARS Coronavirus 2"[tiab] OR "Severe Acute Respiratory Syndrome CoV 2"[tiab] OR "Wuhan coronavirus"[tiab] OR "Wuhan seafood market pneumonia virus"[tiab] OR "SARS2"[tiab] OR "2019-nCoV"[tiab] OR "hcov-19"[tiab] OR „novel 2019 coronavirus"[tiab] OR "2019 novel coronavirus*"[tiab] OR „novel coronavirus 2019*"[tiab] OR "2019 novel human coronavirus*"[tiab] OR „human coronavirus 2019"[tiab] OR "coronavirus disease-19"[tiab] OR "corona virus disease-19"[tiab] OR "coronavirus disease 2019"[tiab] OR "corona virus disease 2019"[tiab] OR "2019 coronavirus disease"[tiab] OR "2019 corona virus disease"[tiab] OR „novel coronavirus disease 2019"[tiab] OR „novel coronavirus infection 2019"[tiab] OR "new coronavirus*"[tiab] OR "coronavirus outbreak"[tiab] OR "coronavirus epidemic"[tiab] OR "coronavirus pandemic"[tiab] OR "pandemic of coronavirus"[tiab] AND ("2019/12/01"[PDAT] : "2099/12/31"[PDAT]) | OR | (("wuhan"[tiab] or china[tiab] or hubei[tiab]) AND ("Severe Acute Respiratory Syndrome Coronavirus 2"[Supplementary Concept] OR "COVID-19" [Supplementary Concept] OR "covid 19 diagnostic testing"[Supplementary Concept] OR "covid 19 drug treatment"[Supplementary Concept] OR "covid 19 serotherapy"[Supplementary Concept] OR "covid 19 vaccine"[Supplementary Concept] OR "coronavirus*"[tiab] OR "corona virus*"[tiab] OR ncov[tiab] OR covid*[tiab] OR sars*[tiab]))) | AND | (((((((((((((reinfection) OR (re-infection)) OR (Re infection)) OR (reactivated)) OR (recurrent)) OR (recurrence)) OR (secondary infections)) OR (reactivation)) OR (relapse)) OR (post-infection)) OR (re-positive)) OR (re positive)) OR (repositive)) |
| --- | --- | --- | --- | --- | --- |
| Embase | ('severe acute respiratory syndrome coronavirus 2':ti,ab OR 'severe acute respiratory syndrome coronavirus 2'/exp OR 'covid 19'/exp OR ncov*:ti,ab OR covid*:ti,ab OR 'sars cov 2':ti,ab OR 'sars-cov-2':ti,ab OR 'sars coronavirus 2':ti,ab OR 'sars coronavirus 2'/exp OR 'severe acute respiratory syndrome cov 2':ti,ab OR 'wuhan coronavirus':ti,ab OR 'wuhan seafood market pneumonia virus':ti,ab OR sars2:ti,ab OR '2019-ncov':ti,ab OR 'hcov-19':ti,ab OR 'novel 2019 coronavirus':ti,ab OR '2019 novel coronavirus*':ti,ab OR 'novel coronavirus 2019'/exp OR '2019 novel human coronavirus*':ti,ab OR 'human coronavirus 2019':ti,ab OR 'coronavirus disease-19':ti,ab OR 'corona virus disease-19':ti,ab OR 'coronavirus disease 2019':ti,ab OR 'coronavirus disease 2019'/exp OR 'corona virus disease 2019':ti,ab OR '2019 coronavirus disease':ti,ab OR 'novel coronavirus 2019*':ti,ab OR 'novel coronavirus disease 2019':ti,ab OR 'novel coronavirus infection 2019':ti,ab OR '2019 corona virus disease':ti,ab OR 'new coronavirus*':ti,ab OR 'coronavirus outbreak':ti,ab OR 'coronavirus epidemic':ti,ab OR 'coronavirus pandemic':ti,ab OR 'pandemic of coronavirus':ti,ab OR 'severe acute respiratory syndrome coronavirus 2 vaccine'/exp OR 'covid 19 vaccine'/exp AND 2020:py) | OR | (wuhan:ti,ab OR china:ti,ab OR hubei:ti,ab) AND ('severe acute respiratory syndrome coronavirus 2':ti,ab OR 'severe acute respiratory syndrome coronavirus 2'/exp OR 'severe acute respiratory syndrome coronavirus 2' OR 'covid*':ti,ab OR 'covid 19'/exp OR 'covid 19' OR coronavirus*:ti,ab OR 'corona virus*':ti,ab OR ncov:ti,ab OR covid*:ti,ab OR sars*:ti,ab OR 'sars coronavirus 2'/exp) | AND | (reinfection:ab,ti OR 're infection':ab,ti OR reactivation:ab,ti OR relapse:ab,ti OR 'secondary infection':ab,ti OR 're positive':ab,ti) AND recurrence:ab,ti |
