# Supplementary material for: Recurrent SARS-CoV-2 infections and their potential risk to public health – a systematic review
Source: PLoS One. 2021 Dec 9;16(12):e0261221. doi: 10.1371/journal.pone.0261221 (PMC8659325; doi:10.1371/journal.pone.0261221)
Supplement: S2 Table — (DOCX) [file pone.0261221.s003.docx]

**Table S 2. Characteristics of SARS-COV-2 re-positive cases as reported in case studies**

| **Author** | **Country** | **Number of cases** | **Age** | **Co-morbidities (Number with co-morbidity)** | **Weeks between episodes** | **Transmission onward** | **Symptomatology of onward cases** | **new RKI definition** | **Reason** |
| --- | --- | --- | --- | --- | --- | --- | --- | --- | --- |
| [Abdhallah H et al., 2020](https://casereports.bmj.com/content/13/12/e239825) | USA | 1 | 30 | 0 | 4 | NR | NA | Not considered | No negative PCR between episodes and last positive detection less than 3 months before second episode |
| [Abu-Raddad LJ et al., 2020](https://www.ncbi.nlm.nih.gov/pmc/articles/PMC7759339/) | Qatar | 2 | 40-44 | 2 | 8-9 | NR | NA | Not considered | Whole genome sequencing (same strains) |
| [AlFehaidi A et al.,2020](https://www.ncbi.nlm.nih.gov/pmc/articles/PMC7585726/) | Qatar | 1 | 46 | 1 | 12 | NR | NA | Probable | CT value <30 and negative test in between |
| [Alonso FOM et al.,2020](https://www.ncbi.nlm.nih.gov/pmc/articles/pmid/32797634/) | Brazil | 1 | 26 | 0 | 4 | NR | NA | Probable | CT value <30 and negative test in between |
| [Alturaif S et al., 2020](https://www.researchsquare.com/article/rs-86920/v1?redirect=/article/rs-86920) | Saudi Arabia | 2 | 23-59 | 0 | 8-16 | NR | NA | Possible | no information on genetic copies or CT value |
| [Bellanti F et al., 2020](https://www.journalofinfection.com/article/S0163-4453(20)30770-2/fulltext) | Italy | 1 | 91 | 1 | 2 | NR | NA | Not considered | Symptoms did not fully resolve (but negative PCR between episodes) |
| [Bellesso M et al., 2020](http://www.htct.com.br/en-second-covid-19-infection-in-patient-avance-S2531137920313080?newsletter=true&coronavirus) | Brazil | 1 | 76 | 1 | 16 | NR | NA | Not considered | No negative PCR between episodes and last positive detection less than 3 months before second episode |
| [Bentivegna E et al., 2020](https://www.ncbi.nlm.nih.gov/pmc/articles/PMC7300757/) | Italy | 1 | 69 | 1 | 4 | NR | NA | Possible | no information on genetic copies or CT value |
| [Bongiovanni M, 2020](https://www.ncbi.nlm.nih.gov/pmc/articles/PMC7537129/) | Italy | 1 | 48 | 0 | 13 | NR | NA | Possible | no information on genetic copies or CT value |
| [Bonifácio LP et al., 2020](https://www.scielo.br/scielo.php?pid=S0037-86822020000100723&script=sci_arttext) | Brazil | 1 | 24 | 1 | 7 | 1 positive HCW | Headache and hyposmia | Possible | no information on genetic copies or CT value |
| [Cao S et al., 2020](https://assets.researchsquare.com/files/rs-23197/v2/7b7416e1-363f-4a45-8fba-32c8b65e66a0.pdf) | China | 1 | 68 | 1 | 6 | NR | NA | Possible | CT value >30 and negative test in between |
| [Cen Y et al., 2020](https://www.ncbi.nlm.nih.gov/pmc/articles/PMC7275981/pdf/main.pdf) | China | 4 | 33.5 (12-49) | NR | > 1 | NR | NA | 2 possible cases 2 not considered | CT value >30 and negative test in between |
| [Chen D et al., 2020](https://www.sciencedirect.com/science/article/pii/S1201971220301223) | China | 1 | 46 | 0 | 2 | NR | NA | Possible | Number of genetic copies <10^6 |
| [Colson P et al., 2020](https://www.ncbi.nlm.nih.gov/pmc/articles/PMC7666873/) | France | 1 | 70 | 1 | 15 | NR | NA | Confirmed | Whole genome sequencing (different strains) |
| [Copolla A et al., 2020](https://www.frontiersin.org/articles/10.3389/fmed.2020.00531/full) | Italy | 1 | 68 | 1 | 2 | NR | NA | Possible | no information on genetic copies or CT value |
| [Dou C et al., 2020](https://www.sciencedirect.com/science/article/pii/S0168822720305520) | China | 1 | 34 | 0 | 5 | NR | NA | Possible | no information on genetic copies or CT value |
| [Duggan NM et al., 2021](https://www.ncbi.nlm.nih.gov/pmc/articles/PMC7335242/) | USA | 1 | 82 | 1 | 6,5 | NR | NA | Possible | no information on genetic copies or CT value |
| [Fehdi MA et al., 2020](https://www.panafrican-med-journal.com/content/series/35/2/35/full/) | Morocco | 1 | 69 | 0 | 3 | NR | NA | Not considered | Symptoms did not fully resolve (but negative PCR between episodes) |
| [Fu W et al., 2020](https://www.ncbi.nlm.nih.gov/pmc/articles/PMC7267393/) | China | 3 | 36 (34-74) | 2 | 2 | NR | NA | Possible | no information on genetic copies or CT value |
| [Gao G et al., 2020](https://link.springer.com/article/10.1007/s15010-020-01485-6) | China | 1 | 70 | 1 | 12 | NR | NA | Probable | CT value <30 and negative test in between |
| [Geling T et al., 2020](https://www.ncbi.nlm.nih.gov/pmc/articles/PMC7351047/) | China | 1 | 24 | 0 | 1 | NR | NA | Possible | no information on genetic copies or CT value |
| [Goldman JD et al., 2020](https://www.ncbi.nlm.nih.gov/pmc/articles/PMC7523175/) | USA | 1 | 60 | 1 | 20 | NR | NA | Confirmed | Whole genome sequencing (different strains) |
| [Gousseff M et al., 2020](https://www.ncbi.nlm.nih.gov/pmc/articles/PMC7326402/) | France | 11 | 55 (19-91) | 7 | 1 | NR | NA | Not considered | No information negative PCR between episodes but recovered fully from symptoms |
| [Gupta V et al., 2020](https://academic.oup.com/cid/advance-article/doi/10.1093/cid/ciaa1451/5910388?login=true) | India | 2 | 25-28 | 1 | 9-14 | NR | NA | Confirmed | Whole genome sequencing (different strains) |
| [Habibzadeh P et al., 2020](https://www.biochemia-medica.com/en/journal/30/3/10.11613/BM.2020.030401/fullArticle) | Iran | 9 | 52 (30-56) | 5 | 3 - 7 | NR | NA | Not considered | No negative PCR and < 3months between episodes |
| [Hanif M et al., 2020](https://www.ncbi.nlm.nih.gov/pmc/articles/PMC7689968/) | Pakistan | 1 | 58 | 0 | 8 | NR | NA | Possible | no information on genetic copies or CT value |
| [Harrington D et al., 2021](https://academic.oup.com/cid/advance-article/doi/10.1093/cid/ciab014/6076528) | UK | 1 | 78 | 1 | 32 | NR | NA | Confirmed | Whole genome sequencing (different strains) |
| [He F et al., 2020](https://link.springer.com/article/10.1007/s10067-020-05230-0) | China | 1 | 39 | 1 | 8 | NR | NA | Possible | no information on genetic copies or CT value |
| [Hu R et al., 2020](https://jamanetwork.com/journals/jamanetworkopen/fullarticle/2766379) | China | 11 | 27 (4-58) | 3 | 3 - 5 | NR | NA | Possible | no information on genetic copies or CT value |
| [Huang J et al., 2020](https://www.nature.com/articles/s41598-020-75629-x) | China | 69 | n=23:0–29yrs,  n=34: 30-54yrs,  n= 12: 54-86yrs | 22 | 3 - 6 | NR | NA | Possible | no information on genetic copies or CT value |
| [Hussein NR et al., 2020](https://sites.kowsarpub.com/jkums/articles/111454.html) | Iraq | 1 | 46 | NR | 7 | NR | NA | Possible | no information on genetic copies or CT value |
| [Islam MJ et al., 2020](https://www.banglajol.info/index.php/BIRDEM/article/view/50994) | Bangladesh | 3 | 54 (28-76) | 2 | 4-17 | NR | NA | Possible | no information on genetic copies or CT value |
| [Jain A et al., 2020](https://pubmed.ncbi.nlm.nih.gov/33295221/) | India | 1 | 21 | 0 | 7 | NR | NA | Possible | no information on genetic copies or CT value |
| [Jian M et al., 2020](https://www.ncbi.nlm.nih.gov/pmc/articles/PMC7151420/) | China | 6 | 45.2 (30-56) | 1 | 4 | NR | NA | Possible | no information on genetic copies or CT value |
| [Jian W et al., 2020](https://bmcpulmmed.biomedcentral.com/articles/10.1186/s12890-020-01348-8) | China | 2 | 8-46 | 0 | 3-4 | NR | NA | Possible | no information on genetic copies or CT value |
| [Lafaie L et al., 2020](https://agsjournals.onlinelibrary.wiley.com/doi/full/10.1111/jgs.16728?casa_token=e0hIW6AZ2_4AAAAA%3AVCEGBA-zncxMKt9OJsOyRhaP21DybwkJuK_e89f1K5AWgEhRfzjlg5pc9TosYfgYKDCZNU5KVTu6MQ) | France | 2 | 84-90 | 2 | 6 | NR | NA | Not considered | 1 case with symptoms not fully resolved (but negative PCR in between) 1 case had no PCR during the first episode |
| [Lancman G et al., 2020](https://jhoonline.biomedcentral.com/articles/10.1186/s13045-020-00968-1) | USA | 1 | 55 | 1 | 4 | NR | NA | Possible | no information on genetic copies or CT value |
| [Lechien JR et al., 2020](https://journals.sagepub.com/doi/10.1177/0145561320970105?url_ver=Z39.88-2003&rfr_id=ori:rid:crossref.org&rfr_dat=cr_pub%20%200pubmed) | France | 2 | 38, 42 | NR | 20-24 | NR | NA | Possible | no information on genetic copies or CT value |
| [Lee JS et al., 2020](https://academic.oup.com/cid/advance-article/doi/10.1093/cid/ciaa1421/5997517?login=true) | South Korea | 6 | 29.5 (17-72) | 3 | 4 – 6 | NR | NA | 1 case confirmed 1 case probable 4 cases possible | 1 whole genome sequencing (different strains) 1 CT < 30 4 CT >30 |
| [Li J et al., 2020](https://www.clinicalmicrobiologyandinfection.com/article/S1198-743X(20)30258-5/fulltext) | China | 1 | 50 | 1 | 5 | NR | NA | Possible | no information on genetic copies or CT value |
| [Li XJ et al., 2020](https://ccforum.biomedcentral.com/articles/10.1186/s13054-020-02877-8) | China | 1 | 41 | NR | 4 | NR | NA | Not considered | No negative PCR between episodes and last positive detection less than 3 months before second episode |
| [Liu F et al., 2020](https://www.ncbi.nlm.nih.gov/pmc/articles/PMC7406422/) | China | 1 | 57 | NR | >1 | NR | NA | Not considered | Symptoms did not fully resolve (but negative PCR between episodes) |
| [Liu F et al.,2020](https://academic.oup.com/femspd/article/78/4/ftaa031/5863936) | China | 1 | 35 | 0 | 4 | NR | NA | Not considered | Symptoms did not fully resolve (but negative PCR between episodes) |
| [Liu Y et al., 2020](https://www.ncbi.nlm.nih.gov/pmc/articles/PMC7711384/) | China | 10 | 40 (20-59) | 4 | 1 - 3 | NR | NA | Possible | no information on genetic copies or CT value |
| [Loconsole D et al., 2020](https://www.ncbi.nlm.nih.gov/pmc/articles/PMC7228864/) | Italy | 1 | 48 | 0 | 6 | NR | NA | Possible | no information on genetic copies or CT value |
| [Luciani M et al., 2020](https://onlinelibrary.wiley.com/doi/10.1002/jmv.26701) | Italy | 1 | 69 | 1 | 6 | NR | NA | Possible | no information on genetic copies or CT value |
| [Luo A et al., 2020](https://www.ncbi.nlm.nih.gov/pmc/articles/PMC7144858/) | China | 1 | 58 | NR | 3 | NR | NA | Not considered | Symptoms did not fully resolve (but negative PCR between episodes) |
| [Mahajan N.N et al., 2021](https://www.researchgate.net/publication/348096798_Clinical_Presentation_of_Cases_with_SARS-CoV-2_Reinfection_Reactivation) | India | 9 | 36(25-51) | NR | 6-11 | NR | NA | Possible | no information on genetic copies or CT value |
| [Marcev S et al., 2020](https://sciendo.com/article/10.2478/amb-2020-0033) | Bulgaria | 1 | 25 | 0 | 12 | NR | NA | Possible | no information on genetic copies or CT value |
| [Mardani M et al., 2020](https://www.ncbi.nlm.nih.gov/pmc/articles/PMC7376341/) | Iran | 1 | 64 | NR | 3 | NR | NA | Possible | no information on genetic copies or CT value |
| [Mendoza JR et al., 2020](file:///C:\Users\abrokwas\AppData\Local\Microsoft\Windows\INetCache\Content.MSO\COVID-19%20in%20a%20patient%20with%20end-stage%20renal%20disease%20on%20chronic%20in-center%20hemodialysis%20after%20evidence%20of%20SARS-CoV-2%20IgG%20antibodies.%20Reinfection%20or%20inaccuracy%20of%20antibody%20testing) | USA | 1 | 51 | 1 | 4 | NR | NA | Possible | no information on genetic copies or CT value |
| [Mulder M et al., 2020](https://europepmc.org/article/PMC/7665355) | Netherlands | 1 | 89 | 1 | 8 | NR | NA | Confirmed | Whole genome sequencing (different strains) |
| [Nachmias V et al., 2020](https://www.ncbi.nlm.nih.gov/pmc/articles/PMC7528892/) | Israel | 1 | 20 | 0 | 12 | NR | NA | Possible | no information on genetic copies or CT value |
| [Nazir N et al., 2020](http://www.apicareonline.com/index.php/APIC/article/view/1369) | India | 1 | 26 | 0 | 13 | NR | NA | Possible | no information on genetic copies or CT value |
| [Nepal R et al., 2020](https://www.ncbi.nlm.nih.gov/pmc/articles/PMC7775023/) | Nepal | 1 | 31 | 1 | 4 | NR | NA | Possible | no information on genetic copies or CT value |
| [Novoa W et al., 2021](https://www.ncbi.nlm.nih.gov/pmc/articles/PMC7802059/) | Colombia | 1 | 44 | 0 | 16 | NR | NA | Possible | no information on genetic copies or CT value |
| [Ogawa Y et al., 2019](https://bmcresnotes.biomedcentral.com/articles/10.1186/s13104-020-05365-y) | Japan | 1 | NR | NR | 6 | 15 negative HCW | NA | Not considered | Symptoms did not fully resolve (but negative PCR between episodes) |
| [Okar L et al., 2020](https://onlinelibrary.wiley.com/doi/10.1002/ccr3.3682) | Qatar | 1 | 31 | 1 | 8 | NR | NA | Possible | no information on genetic copies or CT value |
| [Ozaras R et al., 2020](https://www.ncbi.nlm.nih.gov/pmc/articles/PMC7670194/) | Turkey | 1 | 23 | 1 | 12 | NR | NA | Possible | CT value > 30 |
| [Patrocinio de Jesus R et al., 2020](https://link.springer.com/article/10.1007/s42399-020-00548-x) | Portugal | 1 | 41 | 0 | 3 | 3 positive family members | Respiratory symptoms | Probable | CT value <30 and negative test in between |
| [Peng J et al., 2020](https://www.ncbi.nlm.nih.gov/pmc/articles/PMC7151314/) | China | 7 | 29-67 | NR | 4 | NR | NA | 6 possible cases 1 not considered case | 6 cases with no information on number of genetic copies or CT value 1 case with only positive PCR in anal swab during second episode |
| Pérez-Lago L et al., 2020 | Spain | 1 | 53 | 1 | 20 | 7 positive family members | Varied symptoms | Not classified | does not meet RKI confirmed reinfection criteria |
| [Prado-Vivar B et al., 2020](https://www.thelancet.com/journals/lancet/article/PIIS1473-3099(20)30910-5/fulltext) | Equador | 1 | 46 | 0 | 9 | NR | NA | Confirmed | Whole genome sequencing (different strains) |
| [Radhakrishnan V, 2020](https://www.ncbi.nlm.nih.gov/pmc/articles/PMC7646029/) | India | 1 | 4 | 1 | 4 | NR | NA | Possible | no information on genetic copies or CT value |
| [Salcin S, 2020](https://www.ncbi.nlm.nih.gov/pmc/articles/PMC7718582/) | USA | 1 | 62 | 1 | 20 | NR | NA | Possible | no information on genetic copies or CT value |
| [Selhorst P et al., 2020](https://www.medrxiv.org/content/10.1101/2020.11.05.20225052v1.full) | Belgium | 1 | 39 | NR | Up to 26 | Unclear | Fever, cough, sore throat | Confirmed | Whole genome sequencing (different strains) |
| [Selvaraj V et al., 2020](http://rimed.org/rimedicaljournal/2020/12/2020-12-24-extra-case-selvaraj.pdf) | USA | 1 | 70 | 1 | 28 | NR | NA | Possible | no information on genetic copies or CT value |
| [Sen MK et al.,2020](https://www.ncbi.nlm.nih.gov/pmc/articles/PMC7752104/) | India | 4 | 42 (18-78) | 2 | 3-6 | NR | NA | Possible | no information on genetic copies or CT value |
| [Shahin M et al., 2020](http://www.publichealthmy.org/ejournal/ojs2/index.php/ijphcs/article/view/1285) | Bangladesh | 2 | 38 | 2 | 1 | NR | NA | Possible | no information on genetic copies or CT value |
| [Sharma R et al., 2020](https://www.ncbi.nlm.nih.gov/pmc/articles/PMC7718490/) | Qatar | 1 | 57 | 1 | 11 | NR | NA | Possible | no information on genetic copies or CT value |
| [Takeda C.V.F, 2020](https://www.ajtmh.org/view/journals/tpmd/103/5/article-p1993.xml) | Brazil | 6 | 43.5 median | 3 | 1,5 | NR | NA | 3 cases possible  3 cases not considered | 3 cases no information on number of genetic copies or virus culture 3 cases no negative PCR and < 3months between episodes |
| [Tao J et al., 2020](https://europepmc.org/article/ppr/ppr122569) | China | 2 | 39-50 | 0 | 1 | NR | NA | Possible | no information on genetic copies or CT value |
| [Tehrani HA et al.,2020](https://www.sciencedirect.com/science/article/pii/S2052297520301827?via%3Dihub#!) | Iran | 1 | 15 | 1 | 4 | NR | NA | Probable | genetic copies >10^6/ml |
| [Tillett RL et al., 2021](https://www.sciencedirect.com/science/article/pii/S1473309920307647) | USA | 1 | 25 | 0 | 6 | NR | NA | Confirmed | Whole genome sequencing (different strains) |
| [To KK et al., 2021](https://academic.oup.com/cid/advance-article/doi/10.1093/cid/ciaa1275/5897019?fbclid=IwAR1X2_fMbsb94IJtCxYEByVXZbE_6n6JMX3Rk_Gu9zgliEo-k2ZFdpjm2pg) | Hong Kong | 1 | 33 | 0 | 10 | NR | NA | Confirmed | Whole genome sequencing (different strains) |
| [Tomassini S et al., 2020](https://www.ncbi.nlm.nih.gov/pmc/articles/PMC7422822/) | UK | 6 | 75.8(49-93) | 5 | 6-13 | NR | NA | Possible | no information on genetic copies or CT value |
| [Trisnawati I et al., 2020](https://www.ncbi.nlm.nih.gov/pmc/articles/PMC7536520/) | Indonesia | 4 | 41.5(36-56) | 3 | 3-9 | NR | NA | Possible | no information on genetic copies or CT value |
| [Van Elslande J et al., 2020](https://academic.oup.com/cid/article-lookup/doi/10.1093/cid/ciaa1330) | Belgium | 1 | 51 | 1 | 12 | NR | NA | Confirmed | Whole genome sequencing (different strains) |
| [Wang P et al.,2020](https://pubmed.ncbi.nlm.nih.gov/32697372/) | China | 1 | 33 | 0 | 5 | 2 negative family members | NA | Possible | CT value > 30 |
| [West J, 2021](https://www.rcpjournals.org/content/clinmedicine/early/2020/12/08/clinmed.2020-0912) | UK | 1 | 25 | 0 | 24 | NR | NA | Possible | no information on genetic copies or CT value |
| [Wu J et al., 2020](https://www.ncbi.nlm.nih.gov/pmc/articles/PMC7664131/) | China | 6 | 46(32-71) | 2 | <1 - 3 | NR | NA | Possible | CT value > 30 |
| [Xin H et al., 2020](https://www.researchsquare.com/article/rs-32813/latest.pdf) | China | 1 | 19 | 1 | 6 | Negative family members | NA | Possible | no information on genetic copies or CT value |
| [Yadav SP et al., 2020](https://www.authorea.com/doi/full/10.22541/au.159986505.57940176) | India | 2 | 3 -14 | 2 | 4-6 | NR | NA | Probable | CT value <30 and negative test in between |
| [Yoo SY et al.](https://www.ncbi.nlm.nih.gov/pmc/articles/PMC7276786/) | China | 1 | 8 | 0 | 5 | NR | NA | Possible | no information on genetic copies or CT value |
| [Zhang B et al., 2020](https://www.ncbi.nlm.nih.gov/pmc/articles/PMC7177113/) | China | 7 | 29.5 (13,-35) | 1 | 3 - 4 | NR | NA | Possible | no information on genetic copies or CT value |
| [Zhang R et al., 2020](https://www.frontiersin.org/articles/10.3389/fmed.2020.585485/full) | China | 4 | 36-55 | NR | 18-25 | NR | NA | Possible | no information on genetic copies or CT value |
| [Zheng KI et al., 2020](https://www.ncbi.nlm.nih.gov/pmc/articles/PMC7169645/) | China | 3 | 23-57 | 0 | 2 | NR | NA | Possible | no information on genetic copies or CT value |
| [Zheng SL et al., 2020](https://pubmed.ncbi.nlm.nih.gov/33275266/) | China | 11 | 40.5 (24-56) | NR | 2 | NR | NA | Possible | no information on genetic copies or CT value |
| [Zhou X et al., 2020](https://bmcinfectdis.biomedcentral.com/articles/10.1186/s12879-020-05231-z) | China | 1 | 40 | 0 | 4 | NR | NA | Not considered | Symptoms did not fully resolve (but negative PCR between episodes) |
