# Supplementary material for: Recurrent SARS-CoV-2 infections and their potential risk to public health – a systematic review
Source: PLoS One. 2021 Dec 9;16(12):e0261221. doi: 10.1371/journal.pone.0261221 (PMC8659325; doi:10.1371/journal.pone.0261221)
Supplement: S3 Table — (DOCX) [file pone.0261221.s004.docx]

**Table S 3. Critical appraisal of case reports included**

| **Author** | **Were patient’s demographic characteristics clearly described?** | **Was the patient’s history clearly described and presented as a timeline?** | **Was the first COVID episode clearly described?** | **Were diagnostic tests or assessment methods and the results clearly described?** | **The recovery status of the patient is clearly described?** | **Was the second COVID episode clearly described?** | **Was the intervention and treatment procedures clearly described?** | **Were adverse events or unanticipated events identified and described?** | **Does the case report provide takeaway lessons?** | **Rating** |
| --- | --- | --- | --- | --- | --- | --- | --- | --- | --- | --- |
| [Abdhallah H et al., 2020](https://casereports.bmj.com/content/13/12/e239825) | 1 | 1 | 1 | 1 | 1 | 1 | 1 | NA | 1 | 8 |
| [AlFehaidi A et al.,2020](https://www.ncbi.nlm.nih.gov/pmc/articles/PMC7585726/) | 1 | 1 | 1 | 1 | 1 | 1 | 1 | NA | 1 | 8 |
| [Alonso FOM et al.,2020](https://www.ncbi.nlm.nih.gov/pmc/articles/pmid/32797634/) | 1 | 1 | 1 | 1 | 1 | 1 | 0 | NA | 1 | 7 |
| [Alturaif S et al., 2020](https://www.researchsquare.com/article/rs-86920/v1?redirect=/article/rs-86920) | 1 | 1 | 1 | 1 | 1 | 1 | 1 | NA | 1 | 8 |
| [Artega-Livias K et al., 2020](https://pubmed.ncbi.nlm.nih.gov/33307196/) | 1 | 1 | 1 | 1 | 1 | 1 | 0 | NA | 1 | 7 |
| [Bellanti F et al., 2020](https://www.journalofinfection.com/article/S0163-4453(20)30770-2/fulltext) | 1 | 1 | 1 | 1 | 1 | 1 | 0 | NA | 1 | 7 |
| [Bellesso M et al., 2020](http://www.htct.com.br/en-second-covid-19-infection-in-patient-avance-S2531137920313080?newsletter=true&coronavirus) | 1 | 1 | 1 | 1 | 1 | 1 | 0 | NA | 1 | 7 |
| [Bentivegna E et al., 2020](https://www.ncbi.nlm.nih.gov/pmc/articles/PMC7300757/) | 1 | 1 | 1 | 1 | 1 | 1 | 0 | NA | 1 | 7 |
| [Bongiovanni M, 2020](https://www.ncbi.nlm.nih.gov/pmc/articles/PMC7537129/) | 1 | 1 | 1 | 1 | 1 | 1 | 0 | NA | 1 | 7 |
| [Bonifácio LP et al., 2020](https://www.scielo.br/scielo.php?pid=S0037-86822020000100723&script=sci_arttext) | 1 | 1 | 1 | 1 | 1 | 1 | 1 | NA | 1 | 8 |
| [Cao S et al., 2020](https://assets.researchsquare.com/files/rs-23197/v2/7b7416e1-363f-4a45-8fba-32c8b65e66a0.pdf) | 1 | 1 | 1 | 1 | 1 | 1 | 0 | NA | 1 | 7 |
| [Cen Y et al., 2020](https://www.ncbi.nlm.nih.gov/pmc/articles/PMC7275981/pdf/main.pdf) | 1 | 1 | 1 | 1 | 1 | 1 | 0 | NA | 1 | 7 |
| [Chen D et al., 2020](https://www.sciencedirect.com/science/article/pii/S1201971220301223) | 0 | 1 | 0 | 1 | 1 | 1 | 0 | NA | 1 | 5 |
| [Colson P et al., 2020](https://www.ncbi.nlm.nih.gov/pmc/articles/PMC7666873/) | 1 | 1 | 1 | 1 | 1 | 1 | 0 | NA | 1 | 7 |
| [Copolla A et al., 2020](https://www.frontiersin.org/articles/10.3389/fmed.2020.00531/full) | 1 | 1 | 1 | 1 | 1 | 1 | 1 | NA | 1 | 8 |
| [Dou C et al., 2020](https://www.sciencedirect.com/science/article/pii/S0168822720305520) | 1 | 1 | 1 | 1 | 1 | 1 | 0 | NA | 1 | 7 |
| [Duggan NM et al., 2021](https://www.ncbi.nlm.nih.gov/pmc/articles/PMC7335242/) | 1 | 1 | 1 | 1 | 1 | 1 | 0 | NA | 1 | 7 |
| [Fehdi MA et al., 2020](https://www.panafrican-med-journal.com/content/series/35/2/35/full/) | 0 | 1 | 1 | 1 | 1 | 1 | 1 | NA | 1 | 7 |
| Fu W et al., 2020 | 1 | 1 | 1 | 1 | 1 | 1 | 0 | NA | 1 | 7 |
| [Gao G et al., 2020](https://link.springer.com/article/10.1007/s15010-020-01485-6) | 1 | 1 | 1 | 1 | 1 | 1 | 0 | NA | 1 | 7 |
| [Geling T et al., 2020](https://www.ncbi.nlm.nih.gov/pmc/articles/PMC7351047/) | 1 | 1 | 1 | 1 | 1 | 1 | 1 | NA | 1 | 8 |
| [Goldman JD et al., 2020](https://www.ncbi.nlm.nih.gov/pmc/articles/PMC7523175/) | 0 | 1 | 1 | 1 | 1 | 1 | 0 | NA | 1 | 7 |
| [Gupta V et al., 2020](https://academic.oup.com/cid/advance-article/doi/10.1093/cid/ciaa1451/5910388?login=true) | 1 | 0 | 1 | 1 | 1 | 1 | 0 | NA | 1 | 6 |
| [Hanif M et al., 2020](https://www.ncbi.nlm.nih.gov/pmc/articles/PMC7689968/) | 1 | 1 | 1 | 1 | 1 | 1 | 1 | NA | 1 | 8 |
| [Harrington D et al., 2021](https://academic.oup.com/cid/advance-article/doi/10.1093/cid/ciab014/6076528) | 1 | 1 | 1 | 1 | 1 | 1 | 0 | NA | 1 | 7 |
| [He F et al., 2020](https://link.springer.com/article/10.1007/s10067-020-05230-0) | 1 | 1 | 1 | 1 | 1 | 1 | 1 | NA | 1 | 8 |
| [Hussein NR et al., 2020](https://sites.kowsarpub.com/jkums/articles/111454.html) | 0 | 1 | 1 | 1 | 1 | 1 | 1 | NA | 1 | 8 |
| [Islam MJ et al., 2020](https://www.banglajol.info/index.php/BIRDEM/article/view/50994) | 1 | 1 | 1 | 1 | 1 | 1 | 0 | NA | 1 | 7 |
| [Jain A et al., 2020](https://pubmed.ncbi.nlm.nih.gov/33295221/) | 1 | 1 | 1 | 1 | 1 | 1 | 0 | NA | 1 | 7 |
| [Jian M et al., 2020](https://www.ncbi.nlm.nih.gov/pmc/articles/PMC7151420/) | 1 | 1 | 1 | 1 | 1 | 1 | 1 | NA | 1 | 8 |
| [Jian W et al., 2020](https://bmcpulmmed.biomedcentral.com/articles/10.1186/s12890-020-01348-8) | 1 | 1 | 1 | 1 | 1 | 1 | 1 | NA | 1 | 8 |
| [Lafaie L et al., 2020](https://agsjournals.onlinelibrary.wiley.com/doi/full/10.1111/jgs.16728?casa_token=e0hIW6AZ2_4AAAAA%3AVCEGBA-zncxMKt9OJsOyRhaP21DybwkJuK_e89f1K5AWgEhRfzjlg5pc9TosYfgYKDCZNU5KVTu6MQ) | 1 | 1 | 1 | 1 | 1 | 1 | 1 | NA | 1 | 9 |
| [Lancman G et al., 2020](https://jhoonline.biomedcentral.com/articles/10.1186/s13045-020-00968-1) | 1 | 1 | 1 | 1 | 1 | 1 | 0 | NA | 1 | 7 |
| [Lechien JR et al., 2020](https://journals.sagepub.com/doi/10.1177/0145561320970105?url_ver=Z39.88-2003&rfr_id=ori:rid:crossref.org&rfr_dat=cr_pub%20%200pubmed) | 0 | 1 | 1 | 1 | 1 | 1 | 0 | NA | 1 | 6 |
| [Li J et al., 2020](https://www.clinicalmicrobiologyandinfection.com/article/S1198-743X(20)30258-5/fulltext) | 1 | 1 | 1 | 1 | 1 | 1 | 1 | NA | 1 | 8 |
| [Li XJ et al., 2020](https://ccforum.biomedcentral.com/articles/10.1186/s13054-020-02877-8) | 1 | 1 | 1 | 0 | 1 | 1 | 0 | NA | 1 | 7 |
| [Liu F et al., 2020](https://www.ncbi.nlm.nih.gov/pmc/articles/PMC7406422/) | 0 | 1 | 1 | 1 | 1 | 1 | 1 | NA | 1 | 7 |
| [Liu F et al.,2020](https://academic.oup.com/femspd/article/78/4/ftaa031/5863936) | 1 | 1 | 1 | 1 | 1 | 1 | 0 | NA | 1 | 7 |
| [Loconsole D et al., 2020](https://link.springer.com/content/pdf/10.1007/s15010-020-01444-1.pdf) | 1 | 1 | 1 | 1 | 1 | 1 | 1 | NA | 1 | 7 |
| [Luciani M et al., 2020](https://onlinelibrary.wiley.com/doi/10.1002/jmv.26701) | 1 | 1 | 1 | 1 | 1 | 1 | 0 | NA | 1 | 7 |
| [Luo A et al., 2020](https://www.ncbi.nlm.nih.gov/pmc/articles/PMC7144858/) | 1 | 1 | 1 | 1 | 1 | 1 | 1 | NA | 1 | 8 |
| [Marcev S et al., 2020](https://content.sciendo.com/configurable/contentpage/journals$002famb$002f47$002f3$002farticle-p38.xml) | 1 | 1 | 1 | 1 | 1 | 1 | 0 | NA | 1 | 7 |
| [Mardani M et al., 2020](https://www.sciencedirect.com/science/article/pii/S2052297520300846#!) | 1 | 1 | 1 | 1 | 1 | 1 | 1 | NA | 1 | 8 |
| [Mendoza JR et al., 2020](https://www.sciencedirect.com/science/article/pii/S2214250920302511) | 1 | 1 | 0 | 1 | 0 | 1 | 1 | NA | 1 | 6 |
| [Mulder M et al., 2020](https://europepmc.org/article/PMC/7665355) | 1 | 1 | 1 | 1 | 1 | 1 | 0 | NA | 1 | 7 |
| [Nachmias V et al., 2020](https://www.sciencedirect.com/science/article/pii/S221425092030278X) | 1 | 1 | 1 | 1 | 1 | 1 | 0 | NA | 1 | 7 |
| [Nazir N et al., 2020](http://www.apicareonline.com/index.php/APIC/article/view/1369) | 0 | 1 | 1 | 1 | 1 | 1 | 1 | NA | 1 | 7 |
| [Nepal R et al., 2020](https://www.ncbi.nlm.nih.gov/pmc/articles/PMC7775023/) | 1 | 1 | 1 | 1 | 1 | 1 | 0 | NA | 1 | 7 |
| [0voa W et al., 2021](https://www.ncbi.nlm.nih.gov/pmc/articles/PMC7802059/) | 1 | 1 | 1 | 1 | 1 | 1 | 1 | NA | 1 | 8 |
| [Ogawa Y et al., 2019](https://bmcresnotes.biomedcentral.com/articles/10.1186/s13104-020-05365-y) | 1 | 1 | 1 | 1 | 1 | 1 | 1 | NA | 1 | 8 |
| [Okar L et al., 2020](https://onlinelibrary.wiley.com/doi/10.1002/ccr3.3682) | 1 | 1 | 1 | 0 | 1 | 1 | 1 | NA | 1 | 7 |
| [Ozaras R et al., 2020](https://www.ncbi.nlm.nih.gov/pmc/articles/PMC7670194/) | 1 | 1 | 1 | 1 | 1 | 1 | 1 | NA | 1 | 8 |
| [Patrocinio de Jesus R et al., 2020](https://link.springer.com/article/10.1007/s42399-020-00548-x) | 0 | 1 | 1 | 1 | 1 | 1 | 0 | NA | 1 | 7 |
| [Peng J et al., 2020](https://www.ncbi.nlm.nih.gov/pmc/articles/PMC7151314/) | 0 | 1 | 1 | 1 | 1 | 1 | 0 | NA | 1 | 6 |
| Pérez-Lago L et al., 2020 | 1 | 1 | 1 | 1 | 1 | 1 | 0 | NA | 1 | 7 |
| [Prado-Vivar B et al., 2020](https://www.thelancet.com/journals/lancet/article/PIIS1473-3099(20)30910-5/fulltext) | 0 | 1 | 1 | 1 | 1 | 1 | 0 | NA | 1 | 7 |
| [Radhakrishnan V, 2020](https://www.ncbi.nlm.nih.gov/pmc/articles/PMC7646029/) | 1 | 1 | 1 | 1 | 1 | 1 | 1 | NA | 1 | 8 |
| [Salcin S, 2020](https://www.ncbi.nlm.nih.gov/pmc/articles/PMC7718582/) | 1 | 1 | 1 | 1 | 1 | 1 | 1 | NA | 1 | 8 |
| [Selhorst P et al., 2020](https://www.medrxiv.org/content/10.1101/2020.11.05.20225052v1.full) | 1 | 1 | 1 | 1 | 1 | 0 | 0 | NA | 1 | 6 |
| [Selvaraj V et al., 2020](http://rimed.org/rimedicaljournal/2020/12/2020-12-24-extra-case-selvaraj.pdf) | 0 | 1 | 1 | 1 | 1 | 1 | 1 | NA | 1 | 7 |
| [Shahin M et al., 2020](http://www.publichealthmy.org/ejournal/ojs2/index.php/ijphcs/article/view/1285) | 1 | 1 | 1 | 1 | 1 | 1 | 1 | NA | 1 | 8 |
| [Sharma R et al., 2020](https://www.ncbi.nlm.nih.gov/pmc/articles/PMC7718490/) | 1 | 1 | 1 | 1 | 1 | 1 | 1 | NA | 1 | 8 |
| [Takeda C.V.F, 2020](https://www.ajtmh.org/view/journals/tpmd/103/5/article-p1993.xml) | 1 | 1 | 1 | 1 | 1 | 1 | 1 | NA | 1 | 8 |
| [Tao J et al., 2020](https://europepmc.org/article/ppr/ppr122569) | 1 | 1 | 1 | 1 | 1 |  | 1 | NA | 1 | 7 |
| [Tehrani HA et al.,2020](https://www.sciencedirect.com/science/article/pii/S2052297520301827?via%3Dihub#!) | 1 | 1 | 1 | 1 | 1 | 1 | 1 | NA | 1 | 8 |
| [Tillett RL et al., 2021](https://www.sciencedirect.com/science/article/pii/S1473309920307647) | 1 | 1 | 1 | 1 | 1 | 1 | 1 | NA | 1 | 8 |
| [To KK et al., 2021](https://academic.oup.com/cid/advance-article/doi/10.1093/cid/ciaa1275/5897019?fbclid=IwAR1X2_fMbsb94IJtCxYEByVXZbE_6n6JMX3Rk_Gu9zgliEo-k2ZFdpjm2pg) | 1 | 1 | 1 | 1 | 1 | 1 | 1 | NA | 1 | 8 |
| [Van Elslande J et al., 2020](https://academic.oup.com/cid/article-lookup/doi/10.1093/cid/ciaa1330) | 1 | 1 | 1 | 1 | 1 | 1 | 0 | NA | 1 | 7 |
| [Wang P et al.,2020](https://pubmed.ncbi.nlm.nih.gov/32697372/) | 0 | 1 | 1 | 1 | 1 | 1 | 0 | NA | 1 | 6 |
| [West J, 2021](https://www.rcpjournals.org/content/clinmedicine/early/2020/12/08/clinmed.2020-0912) | 1 | 1 | 1 | 1 | 1 | 1 | 0 | NA | 1 | 7 |
| [Xin H et al., 2020](https://www.researchsquare.com/article/rs-32813/latest.pdf) | 1 | 1 | 1 | Unclear | 1 | 1 | 1 | NA | 1 | 7 |
| [Yadav SP et al., 2020](https://www.authorea.com/doi/full/10.22541/au.159986505.57940176) | 1 | 1 | 1 | 1 | 1 | 1 | 0 | NA | 1 | 7 |
| [Yoo SY et al.](https://www.ncbi.nlm.nih.gov/pmc/articles/PMC7276786/) | 1 | 1 | 1 | 1 | 1 | 1 | 0 | NA | 1 | 7 |
| [Zhang R et al., 2020](https://www.frontiersin.org/articles/10.3389/fmed.2020.585485/full) | 0 | 1 | 1 | 1 | 0 | 1 | 1 | NA | 1 | 6 |
| [Zhou X et al., 2020](https://bmcinfectdis.biomedcentral.com/articles/10.1186/s12879-020-05231-z) | 1 | 1 | 1 | 1 | 1 | 1 | 1 | NA | 1 | 8 |
