# Supplementary material for: Recurrent SARS-CoV-2 infections and their potential risk to public health – a systematic review
Source: PLoS One. 2021 Dec 9;16(12):e0261221. doi: 10.1371/journal.pone.0261221 (PMC8659325; doi:10.1371/journal.pone.0261221)
Supplement: S4 Table — (DOCX) [file pone.0261221.s005.docx]

**Table S 4. Critical appraisal of case series included**

| **Author** | **Were there clear criteria for inclusion in the case series?** | **Was the condition measured in a standard, reliable way for all participants included?** | **Were valid methods used for identification of the condition for all participants included?** | **Did the case series have consecutive inclusion of participants?** | **Did the case series have complete inclusion of participants?** | **Was there clear reporting of the demographics of the participants in the study?** | **Was there clear reporting of clinical information of the participants?** | **Were the outcomes or follow up results of cases clearly reported?** | **Was there clear reporting of the site(s)/clinic(s) demographic information?** | **Was statistical analysis appropriate** | **Rating** |
| --- | --- | --- | --- | --- | --- | --- | --- | --- | --- | --- | --- |
| [Abu-Raddad LJ et al., 2020](https://www.ncbi.nlm.nih.gov/pmc/articles/PMC7759339/) | 0 | 1 | 1 | 0 | 0 | 1 | 1 | 1 | 1 | NA | 6 |
| [Gousseff M et al., 2020](https://www.sciencedirect.com/science/article/pii/S0163445320304540?via%3Dihub) | 0 | 1 | 1 | 1 | 1 | 1 | 1 | 1 | 1 | 0 | 8 |
| [Habibzadeh P eta., 2020](https://www.biochemia-medica.com/en/journal/30/3/10.11613/BM.2020.030401/fullArticle) | 0 | 1 | 1 | 1 | 1 | 1 | 1 | 1 | 1 | 1 | 9 |
| [Hu R et al. 2020](https://jamanetwork.com/journals/jamanetworkopen/fullarticle/2766379) | 1 | 1 | 1 | 1 | 1 | 1 | 1 | 1 | 1 | 1 | 10 |
| [Huang J et al., 2020](https://www.nature.com/articles/s41598-020-75629-x) | 1 | 1 | 1 | 1 | 1 | 1 | 1 | 1 | 1 | 1 | 10 |
| [Lee JS et al., 2020](https://academic.oup.com/cid/advance-article/doi/10.1093/cid/ciaa1421/5997517?login=true) | 1 | 1 | 1 | 0 | 0 | 1 | 1 | 1 | 1 | 1 | 8 |
| [Liu Y et al. 2020](https://www.ncbi.nlm.nih.gov/pmc/articles/PMC7711384) | 1 | 1 | 1 | 1 | 1 | 1 | 1 | 1 | 1 | 1 | 10 |
| [Mahajan N.N et al., 2021](https://www.japi.org/x2747434/clinical-presentation-of-cases-with-sars-cov-2-reinfectionreactivation) | 0 | 1 | 1 | 0 | 0 | 1 | 1 | 1 | 1 | 1 | 7 |
| [Sen MK et al.,2020](https://www.ncbi.nlm.nih.gov/pmc/articles/PMC7752104/) | 1 | 1 | 1 | 0 | 0 | 1 | 1 | 1 | 1 | 0 | 7 |
| [Tomassini S et al., 2020](https://www.ncbi.nlm.nih.gov/pmc/articles/PMC7422822/) | 1 | 1 | 1 | 0 | 0 | 1 | 1 | 1 | 1 | 0 | 7 |
| [Trisnawati I et al., 2020](https://www.ncbi.nlm.nih.gov/pmc/articles/PMC7536520/) | 1 | 1 | 1 | 1 | 1 | 1 | 1 | 1 | 0 | NA | 8 |
| [Wu J et al., 2020](https://www.ncbi.nlm.nih.gov/pmc/articles/PMC7664131/) | 1 | 1 | 1 | 1 | 1 | 1 | 1 | 1 | 1 | 1 | 10 |
| [Zhang B et al., 2020](https://www.ncbi.nlm.nih.gov/pmc/articles/PMC7177113/) | 1 | 1 | 1 | 1 | 1 | 1 | 1 | 1 | Unclear | 1 | 9 |
| [Zheng KI et al., 2020](https://www.ncbi.nlm.nih.gov/pmc/articles/PMC7169645/) | 1 | 1 | 1 | 1 | 1 | 1 | 0 | 1 | 0 | 0 | 7 |
| [Zheng SL et al., 2020](https://pubmed.ncbi.nlm.nih.gov/33275266/) | 1 | 1 | 1 | 1 | 1 | 1 | 1 | 1 | 1 | 1 | 10 |
