# Supplementary material for: Recurrent SARS-CoV-2 infections and their potential risk to public health – a systematic review
Source: PLoS One. 2021 Dec 9;16(12):e0261221. doi: 10.1371/journal.pone.0261221 (PMC8659325; doi:10.1371/journal.pone.0261221)
Supplement: S5 Table — (DOCX) [file pone.0261221.s006.docx]

| **Table S 5. Critical appraisal of observational studies included** | | | | | | | | | | | | | | | |
| --- | --- | --- | --- | --- | --- | --- | --- | --- | --- | --- | --- | --- | --- | --- | --- |
| **Author** | **1. Was the research question or objective clearly stated?** | **2. Was the study population clearly specified and defined?** | **3. Was the participation rate at least 50%?** | **4. Were all subjects selected from similar populations? Were inclusion and exclusion criteria prespecified and applied uniformly?** | **5. Was a sample size justification, power description, or variance and effect estimates provided?** | **6. Were the exposure of interest measured prior to the outcome being measured?** | **7. Adapted: was there confirmed recovery before suspected/confirmed reinfection?** | **8. For exposures that can vary in amount or level, did the study examine different levels of the exposure as related to the outcome?** | **9. Were the exposure measures clearly defined, valid, reliable, and implemented consistently?** | **10. Was the exposure(s) assessed more than once over time?** | **11. Were the outcome measures clearly defined, valid, reliable, and implemented consistently?** | **12. Were the outcome assessors blinded to the exposure status of participants?** | **13. Was loss to follow-up after baseline 20% or less?** | **14. Were key potential confounding variables measured and adjusted statistically?** | **Rating** |
| Abdullah MS et al., 2021 | 0 | 1 | unclear | 1 | 1 | 1 | 1 | NA | 1 | NA | 1 | 0 | 1 | 0 | 8 |
| Abu-Raddad LJA et al., 2020 | 1 | 1 | 1 | 1 | 1 | 1 | unclear | NA | 1 | NA | 1 | 0 | 1 | 0 | 9 |
| Ali AM et al., 2020 | 1 | 1 | unclear | 1 | unclear | 1 | unclear | NA | 1 | NA | 1 | 0 | 1 | 0 | 7 |
| An J et al., 2021 | unclear | 1 | unclear | 1 | unclear | 1 | 1 | NA | 1 | NA | 1 | 0 | 1 | 0 | 7 |
| Bongiovanni M et al., 2020 | 1 | 1 | 1 | 1 | 1 | 1 | 1 | NA | 1 | NA | 1 | 0 | 1 | 0 | 10 |
| Chen J et al., 2020 | 1 | 1 | unclear | 1 | unclear | 1 | 1 | NA | 1 | NA | 1 | 0 | 1 | 1 | 9 |
| Chen LZ et al., 2020 | 1 | 1 | 1 | 1 | 1 | 1 | 1 | NA | 1 | NA | 1 | 0 | 1 | 1 | 11 |
| Du HW et al., 2020 | 1 | 1 | 1 | 1 | 1 | 1 | 1 | NA | 1 | NA | 1 | 0 | 1 | 0 | 10 |
| Hanrath AT et al., 2020 | 1 | 1 | 1 | 1 | 1 | 1 | 1 | NA | 1 | NA | 1 | 0 | 1 | 0 | 10 |
| Hansen CH et al., 2021 | 1 | 1 | unclear | 1 | 1 | 1 | 1 | NA | 1 | NA | 1 | 0 | 1 | 1 | 10 |
| Hu J et al., 2020 | 1 | 1 | unclear | 1 | unclear | 1 | 1 | NA | 1 | NA | 1 | 0 | 1 | 0 | 8 |
| Kang YJ et al. 2020 | 0 | unclear | unclear | 1 | 0 | 1 | unclear | NA | 1 | NA | unclear | 0 | unclear | 0 | 3 |
| Li Y et al., 2021 | 1 | 1 | unclear | 1 | 0 | 1 | 1 | NA | 1 | NA | 1 | 0 | 1 | 0 | 8 |
| Liu T et al., 2020 | 1 | 1 | unclear | 1 | 0 | 1 | 1 | NA | 1 | NA | 1 | 0 | 1 | 0 | 8 |
| Lu J et al., 2021 | 1 | 1 | unclear | 1 | unclear | 1 | 1 | NA | 1 | NA | 1 | 0 | 1 | 0 | 8 |
| Lumley SF et al., 2020 | 1 | 1 | unclear | 1 | 1 | 1 | 1 | NA | 1 | NA | 1 | 0 | 1 | 1 | 10 |
| Luo S et al., 2020 | 1 | 1 | unclear | 1 | 0 | 1 | 1 | NA | 1 | NA | 1 | 0 | 1 | 0 | 8 |
| Pan L et al., 2021 | 1 | 1 | 1 | 1 | 0 | 1 | 1 | NA | 1 | NA | 1 | 0 | 1 | 0 | 9 |
| Patwardhan A, 2020 | 1 | 1 | unclear | 1 | 0 | 1 | 1 | NA | 1 | NA | 1 | 0 | 1 | 0 | 8 |
| Peng D et al., 2021 | 1 | 1 | 1 | 1 | 1 | 1 | 1 | NA | 1 | NA | 1 | 0 | 1 | 0 | 10 |
| Pilz et al., 2021 | 1 | 1 | unclear | 1 | 1 | 1 | 1 | NA | 1 | NA | 1 | 0 | 1 | 0 | 9 |
| Qiao XM et al., 2020 | 1 | 1 | unclear | 1 | 0 | 1 | 1 | NA | 1 | NA | 1 | 0 | 1 | 0 | 8 |
| Tao W et al., 2020 | 1 | 1 | 1 | 1 | 1 | 1 | 1 | NA | 1 | NA | 1 | 0 | 1 | 0 | 10 |
| Tian M et al., 2020 | 1 | 1 | 1 | 1 | 1 | 1 | 1 | NA | 1 | NA | 1 | 0 | 1 | 0 | 10 |
| Wang X et al., 2020 | 1 | 1 | 1 | 1 | 1 | 1 | 1 | NA | 1 | NA | 1 | 0 | 1 | 0 | 10 |
| Wong J et al., 2021 | 1 | 1 | 1 | 1 | 1 | 1 | 1 | NA | 1 | NA | 1 | 0 | 1 | 1 | 11 |
| Wu J et al., 2021 | 0 | 1 | 0 | 1 | unclear | 1 | 1 | NA | 1 | NA | 1 | 0 | 1 | 0 | 7 |
| Xiao AT, 2021 | 1 | 1 | unclear | 1 | 0 | 1 | 1 | NA | 1 | NA | 1 | 0 | 1 | 0 | 8 |
| Yang C et al., 2021 | 1 | 1 | 1 | 1 | 1 | 1 | 1 | NA | 1 | NA | 1 | 0 | 1 | 0 | 10 |
| Yuan J et al., 2021 | 0 | 1 | unclear | 1 | 0 | 1 | 1 | NA | 1 | NA | 1 | 0 | 1 | 0 | 7 |
| Zhang K et al., 2020 | 1 | 1 | unclear | 1 | 0 | 1 | 1 | NA | 1 | NA | 1 | 0 | 1 | 0 | 8 |
| Zhang X et al., 2020 | 1 | 1 | unclear | 1 | 0 | 1 | 1 | NA | 1 | NA | 1 | 0 | 1 | 0 | 8 |
| Zhao W et al., 2021 | 1 | 1 | 1 | 1 | 1 | 1 | 1 | NA | 1 | NA | 1 | 0 | 1 | 0 | 10 |
| Zheng J et al., 2020 | 1 | 1 | unclear | 1 | 0 | 1 | 1 | NA | 1 | NA | 1 | 0 | 1 | 1 | 9 |
| Zhou J et al., 2020 | 1 | 1 | 1 | 1 | 1 | 1 | unclear | NA | 1 | NA | 1 | 0 | 1 | 1 | 10 |
