# Supplementary material for: Recurrent SARS-CoV-2 infections and their potential risk to public health – a systematic review
Source: PLoS One. 2021 Dec 9;16(12):e0261221. doi: 10.1371/journal.pone.0261221 (PMC8659325; doi:10.1371/journal.pone.0261221)
Supplement: S6 Table — (DOCX) [file pone.0261221.s007.docx]

**Table S 6. Critical appraisal of reviews included**

| **Author** | **Is the review question clearly and explicitly stated?** | **Were the inclusion criteria appropriate for the review question?** | **Was the search strategy appropriate?** | **Were the sources and resources used to search for studies adequate?** | **Were the criteria for appraising studies appropriate?** | **Was critical appraisal conducted by two or more reviewers independently?** | **Were there methods to minimize errors in data extraction?** | **Were the methods used to combine studies appropriate?** | **Was the likelihood of publication bias assessed?** | **Were recommendations for policy and/or practice supported by the data?** | **Were the specific directives for new research appropriate?** | **Rating** |
| --- | --- | --- | --- | --- | --- | --- | --- | --- | --- | --- | --- | --- |
| [Arafkas M et al., 2020](https://onlinelibrary.wiley.com/doi/full/10.1002/jmv.26496) | 1 | 0 | 0 | 1 | 0 | 0 | 0 | 0 | 0 | 1 | 1 | **4** |
| [Azam M et al., 2020](https://www.nature.com/articles/s41598-020-77739-y) | 1 | 1 | 1 | 1 | 1 | 1 | 1 | 1 | 1 | 1 | 1 | **11** |
| [Dao TL et al., 2020](https://link.springer.com/article/10.1007/s10096-020-04088-z) | 1 | 0 | 0 | 0 | 0 | 0 | 0 | 0 |  | 1 | 1 | **3** |
| [Elsayed SM et al., 2020](https://www.ncbi.nlm.nih.gov/pmc/articles/PMC7537484/) | 1 | unclear | 1 | 1 | 1 | 1 | 1 | 1 | 0 | 1 | 1 | **9** |
| [Gidari A et al., 2021](https://link.springer.com/article/10.1007/s10096-020-04057-6) | 1 | 1 | 1 | 1 | 1 | 0 | 0 | 1 | 0 | 1 | 1 | **8** |
| [Hoang R, 2020](https://www.medrxiv.org/content/10.1101/2020.09.05.20189134v1.full.pdf) | 1 | 1 | 1 | 1 | 0 | 0 | 1 | 1 | 0 | 1 | 1 | **8** |
| [Mattiuzzi C et al., 2020](https://www.ncbi.nlm.nih.gov/pmc/articles/PMC7717013/) | 1 | 1 | 1 | 1 | 0 | 0 | 0 | 1 | 1 | 1 | 1 | **8** |
| [Yao MQ et al., 2020](https://link.springer.com/article/10.1631/jzus.B2000304) | 1 | 1 | 1 | 1 | 1 | unclear | 1 | 1 | 1 | 1 | 1 | **9** |
